# The Role of Machine Learning to Detect Occult Neck Lymph Node Metastases in Early‐Stage (T1‐T2/N0) Oral Cavity Carcinomas

**DOI:** 10.1002/hed.28189

**Published:** 2025-05-19

**Authors:** Stefania Troise, Lorenzo Ugga, Maria Esposito, Maria Positano, Andrea Elefante, Serena Capasso, Renato Cuocolo, Raffaele Merola, Umberto Committeri, Vincenzo Abbate, Paola Bonavolontà, Riccardo Nocini, Giovanni Dell’Aversana Orabona

**Affiliations:** ^1^ Maxillofacial Surgery Unit Department of Neurosciences, Reproductive and Odontostomatological Sciences, University of Naples Federico II Naples Italy; ^2^ Department of Advanced Biomedical Sciences University of Naples “Federico II” Naples Italy; ^3^ Department of Medicine, Surgery and Dentistry University of Salerno Baronissi Italy; ^4^ Anesthesia and Intensive Care Medicine, Department of Neurosciences, Reproductive and Odontostomatological Sciences University of Naples Federico II Naples Italy; ^5^ Maxillofacial Surgery Unit University Hospital of Terni Terni Italy; ^6^ Otolaryngology—Head and Neck Surgery Department University and Hospital Trust of Verona Verona Italy

**Keywords:** early‐stage tumor, machine learning, occult neck metastasis, Oral cavity carcinoma, radiomics features

## Abstract

**Objective:**

Oral cavity carcinomas (OCCs) represent roughly 50% of all head and neck cancers. The risk of occult neck metastases for early‐stage OCCs ranges from 15% to 35%, hence the need to develop tools that can support the diagnosis of detecting these neck metastases. Machine learning and radiomic features are emerging as effective tools in this field. Thus, the aim of this study is to demonstrate the effectiveness of radiomic features to predict the risk of occult neck metastases in early‐stage (T1‐T2/N0) OCCs.

**Study Design:**

Retrospective study.

**Setting:**

A single‐institution analysis (Maxillo‐facial Surgery Unit, University of Naples Federico II).

**Methods:**

A retrospective analysis was conducted on 75 patients surgically treated for early‐stage OCC. For all patients, data regarding TNM, in particular pN status after the histopathological examination, have been obtained and the analysis of radiomic features from MRI has been extrapolated.

**Results:**

56 patients confirmed N0 status after surgery, while 19 resulted in pN+. The radiomic features, extracted by a machine‐learning algorithm, exhibited the ability to preoperatively discriminate occult neck metastases with a sensitivity of 78%, specificity of 83%, an AUC of 86%, accuracy of 80%, and a positive predictive value (PPV) of 63%.

**Conclusions:**

Our results seem to confirm that radiomic features, extracted by machine learning methods, are effective tools in detecting occult neck metastases in early‐stage OCCs. The clinical relevance of this study is that radiomics could be used routinely as a preoperative tool to support diagnosis and to help surgeons in the surgical decision‐making process, particularly regarding surgical indications for neck lymph node treatment.

## 1 Introduction

Oral cavity carcinomas (OCCs), constituting about half of all head and neck carcinomas, are the most common single entity and rank globally as the sixth most common neoplasm [1]. These lesions can originate from the lips, tongue, floor of the mouth, palate, gingiva, and buccal mucosa and, in between 25% and 45% of cases, can metastasize to cervical lymph nodes, on the basis of staging, grading, and anatomical site [1, 2, 3].

At the time of diagnosis, only about 10% of patients present clinical nodal metastasis [4]. In most cases, clinically, these metastases are undetected, with a rate of occult metastasis ranging from 15% to 34% [5, 6].

Whereas the lymph node status represents one of the most important prognostic factors, the management of a clinically negative neck becomes an important challenge for the surgeon, especially in early‐stage (T1‐T2) oral cancers. A watchful waiting approach with close clinic‐radiological surveillance can be adopted; otherwise, elective neck dissection or biopsy of sentinel lymph nodes has to be considered. It is crucial to highlight that elective neck dissection can cause complications such as shoulder dysfunction or myofascial pain and all the complications related to a second surgical site; moreover, in most cases (about 70%), the neck dissection results histologically negative, constituting an overtreatment [7].

Sentinel lymph node biopsy (SLNB) has emerged as a valuable technique for identifying metastatic spread in head and neck cancer. This procedure involves detecting the first lymph node(s) that drain the primary tumor site and performing a biopsy allowing for targeted pathological assessment. If metastases are identified in the sentinel node, a neck dissection must be performed.

Sentinel lymph node biopsy (SLNB) presents several limitations, including reduced resolution for small lymph nodes, which can lead to false negatives, and challenges in visualizing deep‐seated nodes, such as those in level IIb or the retropharyngeal region, due to signal attenuation. Additionally, patient‐specific factors, such as anatomical variations, high BMI, or altered lymphatic drainage, can further impact the accuracy of the procedure. One of the main challenges of SLNB is the precise identification of the sentinel lymph node, which is crucial for accurate staging and treatment planning. This limitation can be overcome by incorporating SPECT/CT, a technique that enhances the localization of sentinel nodes, thereby improving surgical precision and patient outcomes. However, the availability of SPECT/CT remains limited in some medical centers due to the requirement for specialized equipment and trained personnel [8, 9, 10, 11].

Hence, the need to identify rapid and reproducible laboratory, clinical, and instrumental parameters capable of detecting occult metastases or at least minimizing the risk of their presence, to establish the most appropriate treatment, avoiding undertreatment/overtreatment and consequent morbidity. Different markers might be useful to estimate the risk of metastases in oral squamous cell carcinoma (OSCC), such as T‐stage, grading, tumor location, thickness of tumor infiltration, degree of differentiation, lympho‐vascular invasion, tumor budding, perineural invasion, depth of invasion (DOI), patient's age, and inflammatory status, in particular Neutrophil‐to‐Lymphocyte Ratio (NLR) [11, 12, 13, 14].

The NLR reflects a highly pro‐inflammatory status and has emerged as a potential indicator of cancer progression and lymph node metastases. Its clinical relevance lies in its accessibility and reproducibility from routine blood cell counts in oncological patients [13, 14].

The biopsy sample can be studied in order to assess PD‐L1 expression, serving as a marker to identify high‐risk tumors for progression [15].

However, these parameters may not always be sufficient to identify individuals at high risk of tumor progression due to the significant variability of results in relation to chronic inflammatory diseases and patient status [16, 17].

In this context, the radiomics, which quantitatively analyzes high‐performance medical images to extract features that describe image contents, is emerging as a valid tool. These radiomic features can be combined with other patient data for the diagnosis and prognosis of head and neck pathologies, using machine learning and artificial intelligence techniques [18, 19, 20, 21].

The objective of this study is to demonstrate the effectiveness of radiomic features in predicting neck lymph node metastases in early‐stage oral cavity carcinomas (T1‐T2) with a clinically negative neck (N0), using machine‐learning algorithms. With the application of this algorithm, a significant improvement in terms of diagnosis and management of OCC is expected, in order to support the surgical decision‐making process. The clinical relevance of this study will be to routinely employ this protocol in the staging of these tumors to guide the diagnostic‐therapeutic process while avoiding the risk of overtreatment/undertreatment.

## 2 Materials and Methods

### 2.1 Study Design

A retrospective analysis was conducted between January 2012 and May 2022 on patients admitted at the Maxillofacial Surgery Unit of the University Federico II of Naples for cT1‐T2/cN0 OCCs. All the data were obtained by the retrospective analysis of patients' medical records.

The diagnostic workup for all the patients involved a complete physical examination, routine blood tests, neck Ultrasonography (US), and Magnetic Resonance Imaging (MRI) of the head and neck, performed no later than 20 days before surgery. All the patients who satisfied these inclusion criteria were enrolled in the study:

− Diagnosis of squamous OCC confirmed by a preoperative incisional biopsy;
− Classified early stage cT1‐T2 OCC with clinically negative neck (cN0), according to preoperative American Joint Committee on Cancer (AJCC) classification (8th edition) [22].
− Clinical histopathological DOI > 3 mm and consequent indication for END;
− No previous/simultaneous cancer at any other site and no previous radiotherapy or chemotherapy;
− Availability of preoperative MRI performed within 20 days before surgery with adequate radiomics features;
− Surgery of tumor excision and selective neck dissection with definitive histologic diagnosis of oral cavity carcinoma; and
− A minimum of 24 months of post‐operative follow‐up.

Patients who did not satisfy the inclusion criteria or met the following criteria were excluded from this study:

− Radiotherapy or chemotherapy in the clinical history;
− Previous/simultaneous tumor in other sites;
− Classified clinical advanced OCC (cT3‐cT4);
− Clinically positive neck (cN+) or presence of extra lymph node metastasis (M+);
− MRI with presence of artifacts or, otherwise, unreadable or not analyzed with machine learning systems; and
− Lost at follow‐up.

The research adhered to the principles outlined in the Declaration of Helsinki, and informed consent was obtained from every involved patient for all diagnostic and surgical procedures and for personal data processing. Given the retrospective nature and the non‐interventional design, the study was waived for ethics committee approval.

### 2.2 Data Collection

The data were collected through patients' medical records, which included demographic information (age, sex), the site of the tumor and the consequent surgical treatment, the pathological tumor grading, and the pathological stage according to the Union for International Cancer Control/American Joint Committee on Cancer tumor–node– metastasis (TNM‐UICC/AJCC) classification (8th edition) [22].

The study involved only patients with early‐stage (T1/T2, cN0) OCC with depth of invasion (DOI) > 3 mm who, according to current literature and NCCN 2022 guidelines [23, 24, 25] were candidates for elective neck dissection (END).

All patients underwent a minimum follow‐up of 24 months, with 6‐monthly clinical and instrumental checks with ultrasound and MRI and an annual total body PET/CT scan.

### 2.3 Image Acquisition and Handcrafted Radiomics

Participants in the study were examined with a 3 T MR scanner (Magnetom Trio, Siemens Medical Solutions, Erlangen, Germany). The MR protocol included a T2‐weighted Turbo Spin Echo sequence on the axial plane (repetition time = 7820 ms; echo time = 75 ms; slice thickness = 3 mm; averages = 3; echo train length = 14; acquisition matrix = 384 × 384; field of view = 200 × 200 mm). A radiologist with 5 years of expertise in head and neck imaging carefully outlined oral cavity cancers by carrying out a three‐dimensional segmentation on the volume of interest (VOI), using ITKSnap v3.8.0 software, a freely available segmentation software. For image pre‐processing and radiomic parameter extraction, a well‐known, open‐source Python tool (Pyradiomics v3.0.1) has been used. Initially, images and VOIs were standardized to a consistent isotropic voxel size of 2 × 2 × 2 mm, essential for further pre‐processing and filter applications. Voxel intensity was then normalized against the mean intensity and standard deviation, with a discretization using a fixed bin width of 3. Both original pre‐processed T2‐weighted images and those processed through filters were used for extracting features. Specifically, this involved a Laplacian of Gaussian filter, with sigma values ranging from 2.0 (for finer textures) to 5.0 (for coarser textures) in 1.0 increments, and a wavelet decomposition filter.

### 2.4 Machine Learning

**FIGURE 1:**
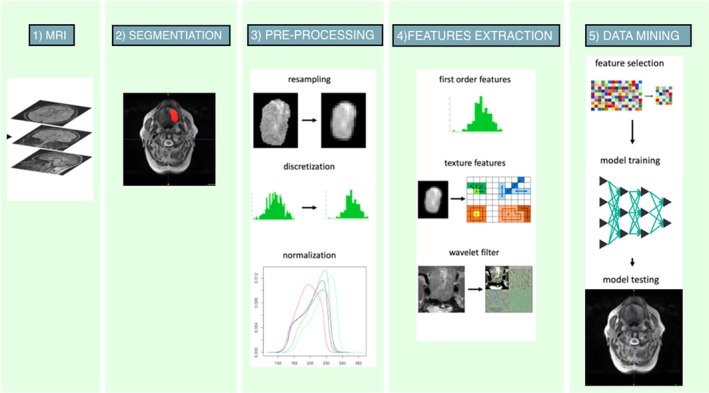
Digital workflow of radiomic features extraction. Step 1: Collect MRI scans; Step 2: Image segmentation on MRI scans; Step 3: Pre‐processing; Step 4: Radiomic features extraction; Step 5: Data mining. [Color figure can be viewed at wileyonlinelibrary.com]

The analysis of the gathered dataset was performed using two open‐source data mining tools: Scikit‐learn and Weka (version 3.8). Two‐thirds of the data set was used for fine‐tuning the hyperparameters, forming the training set, while the remaining third served as a hold‐out set for testing the model on new data. A min‐max scaler was applied to normalize the input features across both training and testing sets, ensuring values ranged between 0 and 1. Features with low variability (variance ≤ 0.01) or high intercorrelation (≥ 0.8) were removed. Anticipating an imbalanced dataset, the Synthetic Minority Oversampling Technique (SMOTE) was utilized on the training set to mitigate this issue. A Gain Ratio analysis helped in identifying the most effective subset of features. These steps prepared the data for training a Random Forest (RF) model, which was validated using 10‐fold cross‐validation. The model's capability to accurately predict lymph node status was then assessed using the test set. The radiomic digital work flow was resumed in Figure 1.

## 3 Results

### 3.1 Population

Considering the inclusion and exclusion criteria, 75 patients were enrolled in the study. All the features of these included patients were resumed in Table 1.

**TABLE 1:** Main features of the 75 enrolled patients.

General population features	
Variables	Total cases 75
Age (years)	
< 60	24 (32%)
> 60	51 (68%)
Gender	
Female	36 (48%)
Male	39 (52%)
Tumor location	
Tongue	36 (48%)
Oral floor	25 (33%)
Inferior alveolar gingiva	8 (11%)
cT status	
cT1	32 (42.7%)
cT2	43 (57.3%)
pT status	
pT1	28 (37%)
pT2	26 (34.6%)
pT3	13 (17.3%)
pT4	8 (11.1%)
p grading	
G1	7 (9.9%)
G2	30 (39.5%)
G3	38 (50.6%)
pN status	
N0	56 (74.7%)
N1	2 (pT1) (2.7%)
	2 (pT2) (2.7%)
	3 (pT3) (4.0%)
N2	2 (pT4) (2.7%)
	1 (pT2) (1.3%)
	3 (pT3) (4.0%)
	3 (pT4) (4.0%)
N3	2 (pT3) (2.7%)
	1 (pT4) (1.3%)

**FIGURE 2:**
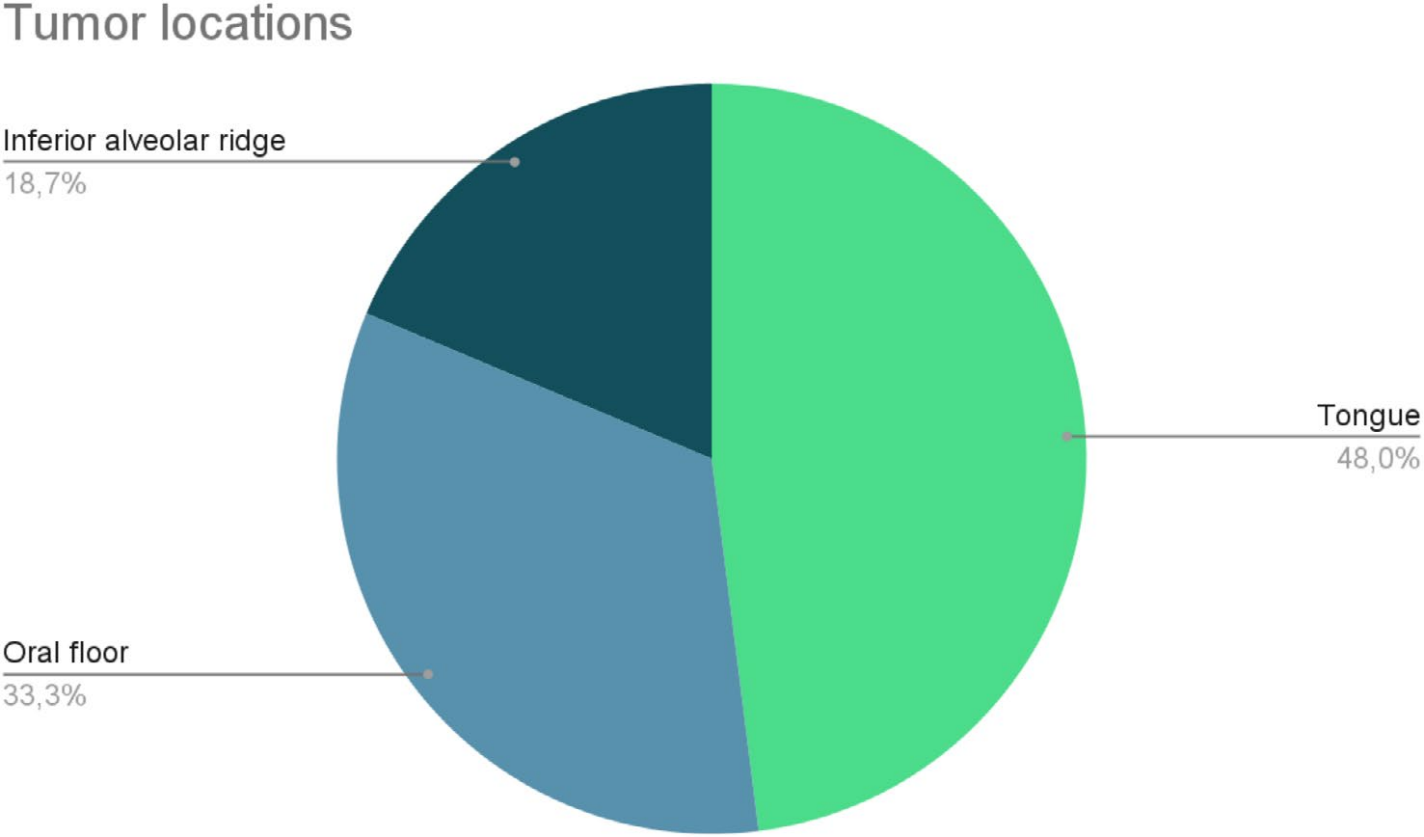
Tumors location. 48% in the tongue, 33.3% in the oral floor, and 18.7% in the alveolar ridge. [Color figure can be viewed at wileyonlinelibrary.com]

The sex of the sample was male for 52% (*n* = 39) and female for 48% (*n* = 36), and an average age at the time of the intervention of 64.8 years (range: 30–94 years). The following data presents a distribution of tumors across various anatomical regions within the oral cavity, derived from a total of 75 cases. The tongue appears to be the most affected, accounting for 48% (36 cases) of the total tumors. Following this, the oral floor represents 33% (25 cases) of the cases, while the inferior alveolar ridge/gingiva contributes for 19% (14 cases). The tumor location rates are resumed in Figure 2.

In all cases, the surgical procedure was an enlarged excision to completely remove the primary OCC with free margins (> 5 mm), and an END.

Regarding the histological grading, the majority of cases, accounting for 50.6% (*n* = 38), were classified as Grade 3 (G3); 39.5% (*n* = 30) of patients were classified as Grade 2 (G2); and 9.9% of patients (*n* = 7) as Grade 1 (G1).

After surgery, regarding the definitive node status, 19/75 (25.3%) patients resulted positive for lymph node metastases (pN + group), while 56/75 (74.7%) patients tested negative (pN0 group). Thus, patients were categorized into pathological stages according to AJCC guidelines: pT1 37% (*n* = 28), pT2 34.6% (*n* = 26), pT3 17.3% (*n* = 13), and pT4 11.1% (*n* = 8).

Patients with coherent clinical and pathological staging were 28/75 pT1 (37.3%) and 22/75 pT2 (34.7%), while in 25/75 patients (33.3%) the clinical staging did not concord to the pathological one, due to the microscopic involvement of tongue muscles and mandibular cortical bone, that classified the tumors as more advanced.

Among pT1 cases, the identification of two lymph node metastases resulted in two cases being staged as N1. Within the pT2 category, two cases were staged as N1, and an additional case was classified as N2. Among the remaining clinical cases, 13 tumors were designated as pT3, of which eight exhibited metastases: three N1, 3 N2 (comprising N2b and N2c), and two N3c. The final eight cases were staged as pT4, with six of them demonstrating metastasis: two N1, three N2, and one N3.

At six‐monthly outpatient checks, up to 24 months of follow‐up, none of the 56 pN0 patients developed neck lymph node metastases.

### 3.2 Image Analysis

In this study, an extensive extraction process identified 1197 texture features, which included shape, first‐order, and higher‐order texture attributes from both original and processed images. For detailed information about these features, readers are invited to refer to the Pyradiomics online documentation, available at https://pyradiomics.readthedocs.io/en/latest/features.html. Among these features, none displayed low variance, but 1030 exhibited high intercorrelation. Through Gain Ratio analysis, five features were pinpointed as most predictive for the study's goal, namely, log‐sigma‐3‐0‐mm‐3D_gldm_DependenceVariance, wavelet‐LLH_glszm_ZoneVariance, wavelet‐HHH_glcm_Idmn, log‐sigma‐4‐0‐mm‐3D_glcm_Idm, and wavelet‐LHL_firstorder_Mean.

The Random Forest (RF) model demonstrated a 72% accuracy rate on the test group, as shown in Table 2, with both sensitivity and specificity rates of 0.7.

**TABLE 2:** Summary of the random forest outputs on the test set.

Correctly classified instances	18 (72%)
Incorrectly classified instances	7 (28%)
Kappa statistic	0.4068
Mean absolute error	0.326
Root mean squared error	0.4492
Total number of instances	25

Comprehensive accuracy details and the confusion matrix can be found in Tables 3 and 4.

**TABLE 3:** Detailed accuracy metrics by class.

	TP rate	FP rate	Precision	Recall	F‐measure	MCC	ROC area	PRC area
N0	0.750	0.33	0.800	0.750	0.774	0.408	0.753	0.816
N+	0.667	0.250	0.600	0.667	0.632	0.408	0.753	0.685
Weighted Avg.	0.720	0.303	0.728	0.720	0.723	0.408	0.753	0.769

Abbreviations: FP, false positives; MCC, Matthews correlation coefficient; N+, presence of lymph node metastases; N0, absence of lymph node metastases; PRC, precision‐recall curve; ROC, receiver operator curve; TP, true positives; Weighted Avg., weighted average.

**TABLE 4:** Confusion matrix for the test group.

		Actual class	
		N0	N+
Predicted class	N0	12	4
	N+	3	6

## 4 Discussion

Neck metastases are the most significant prognostic factor for OCC. Hence, effective management of lymph nodes is crucial for treating this condition. Numerous established protocols are available in the literature for treating positive necks with metastasis, while the management of clinically negative necks is still a debated topic.

Currently, surgeons can choose from several therapeutic alternatives. In patients with early‐stage tumors of the oral cavity, a watchful waiting approach can be adopted; otherwise, elective neck dissection or biopsy of the sentinel lymph node has to be considered.

It's crucial to highlight that the literature reports a complications rate of elective neck dissection in 29% of cases. This approach is burdened by increased morbidity, primarily due to the involvement of a second surgical site. In a substantial majority of cases (70%–80%), this choice may indeed constitute overtreatment [26].

Sentinel lymph node biopsy (SLNB) has emerged as a valuable technique in this context. SLNB involves the identification and biopsy of the first lymph node(s) that drain the primary tumor site. This minimally invasive procedure aids in determining the presence or absence of metastatic spread, allowing for a more targeted and nuanced approach to neck management. If the sentinel lymph node is negative for cancer cells, the likelihood of nodal involvement is considered low, and elective neck dissection may be avoided, reducing unnecessary morbidity. However, if the sentinel node shows evidence of metastasis, appropriate neck dissection must be performed to address potential lymphatic spread. In 2015, the Sentinel European Node Trial (SENT) assessed the identification of metastatic lymph nodes through the sentinel lymph node biopsy on a sample of 415 patients and reported an overall sensitivity and negative predictive value of 86% and 95%, respectively [27].

However, the sensitivity of sentinel lymph node biopsy in the literature ranges from 75% to 100% for detecting the initial draining lymph node in oral cavity squamous carcinomas [28], although identification using SPECT has limitations. These include the limited resolution for small lymph nodes, which may lead to false negatives, as well as the challenge of visualizing deep‐seated nodes, such as those in level IIb or retropharyngeal regions, due to signal attenuation. Another important limitation regards the shine‐through phenomenon, which refers to interference from the primary tumor signal, complicating the identification of the sentinel lymph node. Liao et al. [29] report that this effect is especially problematic when the tumor is near the lymphatic drainage sites. Several authors confirm that this phenomenon can significantly lower the accuracy of the procedure [30, 31]. Strategies to minimize this effect concern the use of multimodal imaging and blue dye alongside radioactive tracers [32]. Additionally, patient‐specific variability, such as anatomical variations, high BMI, or altered lymphatic drainage, can affect the effectiveness of SPECT/CT. Furthermore, the availability of SPECT/CT is limited in some medical centers, as it requires specialized equipment and expertise. Given these constraints, while the authors confirm the diagnostic value of SPECT/CT, they propose radiomics analysis as a complementary tool, particularly in settings where technology and expert healthcare professionals, including radiologists and pathologists, could be restricted [8, 9, 10, 11].

Several Authors have described different prognostic factors in OCC: tumor differentiation, tumor thickness and depth of invasion, perineural invasion, lympho‐vascular invasion, and the inflammatory status [33, 34, 35]. According to the literature, the prevalence of occult neck metastases may range from 15% to 60%, influenced by these different prognostic factors [36, 37].

Currently, the challenge of the surgeon is to find a compromise between the need to correctly assess the metastasis risk and to avoid overtreatment with neck dissection. Hence, there is an urgent need for identifying a reliable predictive tool for preoperative evaluation of metastatic potential to support the surgical decision‐making process.

In recent years, oncological studies have discussed diagnostic approaches based on artificial intelligence, machine learning, and radiomics metrics. Radiomics has shown significant effectiveness in crafting decision‐making support tools, extracting from image scans diagnostic features [38, 39, 40, 41]. One of the pioneering works that contributed to introducing radiomics into the oncology field and occult metastases search was published by Lambin in 2012 [42].

In this study, we chose to focus on radiomics features extracted from the primary tumor rather than from the lymph nodes. This decision was based on the premise that radiomic features derived from the primary tumor can capture intrinsic biological properties, such as tumor heterogeneity, angiogenesis, and cellularity, which are key determinants of metastatic potential. Although radiomics analysis of nodes may provide a more direct assessment of pathologic nodal status, it also presents several challenges, including variability in nodal involvement, anatomical complexity, and the risk of including normal or reactive nodes, which could introduce noise and affect reproducibility. While some studies have explored nodal radiomics, the reliability of such features remains an area of ongoing research. Future studies incorporating both primary tumor and nodal radiomics could provide a more comprehensive approach to predicting nodal metastasis and improve our understanding of tumor biology and metastatic behavior.

Other authors employed radiomics and artificial intelligence in predicting occult metastases in tongue and oral cavity tumors. Kubo et al. [43] in their study described a radiomic protocol to predict occult lymph node metastases in 161 patients with tongue cancer. CT images were transferred to a radiomic platform. The volume of interest (VOI) was the total level of the neck node, including levels Ia, Ib, II, III, and IVa on the ipsilateral side. The dimensionality of radiomic features was reduced using absolute minimum shrinkage and selection operator logistic regression analysis. Five classifiers were compared with or without the synthetic minority oversampling technique (SMOTE). For the analysis at the level of all cervical lymph nodes, Random Forest with SMOTE was the best model, with an accuracy of 0.85 and an AUC score of 0.92. For the analysis at the level of the single lymph node, a support vector machine with SMOTE was the best model, with an accuracy of 0.96 and an AUC score of 0.98.

In Wang et al.'s study [44], the radiomics model was employed to detect lymph nodes metastases in 120 cases of head and neck cancers. The radiomics model, which incorporates radiomic features, apparent diffusion coefficient (ADC), and cervical lymph node size, achieved better performance in predicting cervical lymph node status with an AUC of 0.83.

Alongside radiomics, the effectiveness of Artificial Intelligence (AI), particularly in the context of machine learning, has been demonstrated in supporting surgeons in the management of occult lymph node metastases.

In their 2019 study, Bur et al. [45] included patients who underwent primary tumor resection and elective neck dissection for clinically T1‐2 N0 oral cavity squamous cell carcinoma (OCSCC), as identified from the National Cancer Database (NCDB). Their machine‐learning algorithm's training set comprised 654 patients from the NCDB, while the test set included 71 patients treated at a single academic institution. Performance was assessed using the area under the receiver operating characteristic curve (AUC). The Decision Forest algorithm demonstrated the best classification performance (AUC = 0.840).

Although the topic has been discussed in the literature [46], our study allows us to confirm the effectiveness of machine learning in the detection of occult metastases from OCSCC, considering a more uniform sample. In fact, in our sample, only the oral regions at the highest risk of metastases are considered, and only one type of radiological examination is used for radiomic investigations, the MRI. Therefore, the purpose of this study is to investigate the role of machine‐learning algorithms in the detection of occult lymph node metastases in early‐stage OCSCC.

In accordance with recent literature, our study demonstrated that radiomics, through a machine‐ learning approach, exhibited an effectiveness in preoperatively discriminating potential occult neck metastases in early‐stage OCC. To ensure consistency and avoid potential misinterpretation, model performance is now reported uniformly using AUC, sensitivity, and specificity on the test dataset, which provides a more robust evaluation of generalizability. On the test set, the model achieved an AUC of 0.8, with a sensitivity and a specificity of 0.7. These results indicate good discriminative ability and suggest that the model generalizes well to unseen data.

In this study, we emphasize the importance of negative predictive value (NPV) as a critical performance metric, given its direct clinical implications. A high NPV is essential to minimize false‐negative predictions, which could result in missed diagnoses of metastatic involvement and, consequently, suboptimal treatment planning. Ensuring a low rate of false negatives is particularly important in this context, as it reduces the risk of undertreatment and improves patient outcomes. In addition to NPV, sensitivity is another key metric, as it reflects the model's ability to correctly identify patients with nodal metastases, thereby ensuring appropriate selection for further evaluation or aggressive therapeutic strategies. While other metrics, such as accuracy, specificity, and positive predictive value (PPV), provide complementary insights, our study prioritizes NPV and sensitivity to enhance clinical applicability and decision‐making. Future studies should further optimize these metrics to refine predictive models and improve their reliability in real‐world clinical settings.

Another factor that may have contributed to the slightly lower performance of our model compared to previous studies is the heterogeneity of tumor sites included in our cohort. Specifically, there are inherent differences in anatomical boundaries, shape variability, and tissue interfaces when contouring tumors of the tongue, floor of mouth, and alveolar ridge. These differences can pose additional challenges for automated segmentation algorithms, which may struggle to generalize across such anatomically distinct regions. Future work may benefit from stratifying models based on tumor subsite or incorporating site‐specific features to enhance performance.

Moreover, these results are promising as this digital algorithm could be routinely used to support preoperatively the diagnosis and to guide the surgical decision‐making process.

The clinical relevance of this study is that, using these tools, the surgeon can avoid overtreatment/undertreatment of the neck, also benefiting in terms of healthcare costs [47].

However, our study has several limitations: interpreting algorithm results may not be intuitive and requires specific clinician expertise with a long learning curve; the retrospective nature and the limited sample size. Another parameter to consider is the manual segmentation procedure for lesions and metastases; this limitation should be overcome by employing a semi‐automatic procedure for outlining the regions of interest (ROIs). For all these reasons, this study could be considered a hypothesis‐generator study and further prospective studies need to be conducted to validate this hypothesis.

## 5 Conclusion

Our results seem to show that radiomic features and machine learning algorithms are effective tools in detecting metastatic lymph nodes in early‐stage (T1‐T2) oral cavity carcinomas. Machine learning thus represents a useful tool to guide clinicians in the decision‐making process, especially regarding surgical indications for neck dissection. The key point of the proposed protocol consists in the possibility of using data about radiomic features obtained from MRI to assess the tumor staging and aggressivity and, consequently, to support the diagnosis of correct clinical staging of the patients. The assessment of the correct staging is crucial to guide the treatment, to avoid undertreatment in case of metastases or overtreatment in cases of negative neck. Clearly, these results are to be considered preliminary and further prospective studies need to be conducted to validate this hypothetic protocol.

## Author Contributions

**Stefania Troise and Lorenzo Ugga:** conceptualization and study design. **Maria Esposito and Maria Positano:** writing – original draft. **Andrea Elefante, Serena Capasso and Renato Cuocolo:** data elaboration and analysis. **Raffaele Merola, Umberto Committeri, Vincenzo Abbate, Riccardo Nocini, and Paola Bonavolontà:** writing – review. **Giovanni Dell'Aversana Orabona:** final approval.

## Ethics Statement

Giving the retrospective nature of the study, ethical review and approval were waived by the Ethics Committee for biomedical activities of the University Federico II of Naples.

## Consent

Prior to any diagnostic or therapeutic procedures explicit written consent was obtained from all patients according to the World Medical Association Declaration of Helsinki.

## Conflicts of Interest

The authors declare no conflicts of interest.

## Data Availability

Data sharing is not applicable to this article as no new data were created or analyzed in this study.
